# A Simple Inhaler

**Published:** 1889-08

**Authors:** 


### A Simple Inhaler.

Dr. Ernest E. Maddox gives the following suggestion for making a simple inhaler, in the *Practitioner*, May, 1889. In it such remedies as compound tincture of benzoin, methol, and oil of eucalyptus may be used :

"Coil a piece of paper into the shape of a cigarette, and fix it with gum. Then insert into one end a small uncompressed piece of absorbent cotton-wool, upon which a drop or two of the desired medicament has been poured. Air is now drawn through the tube by the patient, who holds the other end between his lips. This plan is by many patients, especially by men, preferred to the use of any form of respirator, or to inhalations mingled with steam. These last, moreover, have a relaxing effect in some atonic conditions of the throat."

Of a number of remedies, including menthol, inhaled in this way by a patient suffering from pulmonary phthisis, he found that oil of peppermint gave most satisfaction. A small tube of vulcanite flattened like a cigarette-halder at one end, with a raised flange or border to be held within the lips, would doubtless, he says, answer still better; but an inhaler, which when needed can be made on the spot, has advantages of its own.

AN UNAPPRECIATIVE PHYSICIAN.—Under the title: "Good Books and Periodicals *versus* Ignorance and Laziness—a Rich Letter," the *Texas Health Journal*, June, 1889, says: The indifference of some at the current medical and sanitary literature of the age is passingly strange. It is a problem hard to solve. These reflections result from a recent incident incomprehensible in its immensity. A subscriber returned this *Journal* with the following soul-stirring revelation: "Stop the Medical Health—am over red now—all I knead is treatment—got to big a library anyhow—besides I already take the theripudic gazet—dident subscribe for your *Journal* know how. ——, M. D." We are confident this man is over "red"—but just how "red" we have no means of ascertaining—and that he thoroughly "kneads" his treatment—blue mass especially. Being already overstocked with the "theripudic gazet" and "to big a library," he "dident" "knead" the *Journal* "know how"—therefore we feel indisposed to press our claims lest the gentleman become overwhelmed with pure "medical health." Regretfully we leave him to the peaceful enjoyment of his erudition and peculiar vernacular. So long as such men are entrusted with human lives the cause of a high mortality in some localities may, with some degree of certainty, be surmised.

